# Exploring perceptions of meaningfulness in visual representations of bivariate relationships

**DOI:** 10.7717/peerj.6853

**Published:** 2019-05-14

**Authors:** Nataly Beribisky, Heather Davidson, Robert A. Cribbie

**Affiliations:** Department of Psychology, York University, Toronto, Canada

**Keywords:** Effect sizes, Overlapping histograms, Scatterplots, Practical significance

## Abstract

Researchers often need to consider the practical significance of a relationship. For example, interpreting the magnitude of an effect size or establishing bounds in equivalence testing requires knowledge of the meaningfulness of a relationship. However, there has been little research exploring the degree of relationship among variables (e.g., correlation, mean difference) necessary for an association to be interpreted as meaningful or practically significant. In this study, we presented statistically trained and untrained participants with a collection of figures that displayed varying degrees of mean difference between groups or correlations among variables and participants indicated whether or not each relationship was meaningful. The results suggest that statistically trained and untrained participants differ in their qualification of a meaningful relationship, and that there is significant variability in how large a relationship must be before it is labeled meaningful. The results also shed some light on what degree of relationship is considered meaningful by individuals in a context-free setting.

## Introduction

In many instances, researchers need to consider the practical significance of a relationship. This may be necessary when interpreting the magnitude of an effect size or quantifying a minimally important relationship in equivalence testing. Despite this, there has been little research exploring when relationships (e.g., correlation or mean difference) begin to be interpreted as meaningful or practically significant. Due to this lack of information, many researchers have defaulted to using Cohen’s (1988) effect size recommendations that specify lower bounds for interpreting a relationship as “small”, regardless of the context of the study. Even less is known about the variability surrounding the interpretation of effect sizes; judgments of effect sizes may vary greatly from person to person. For example, individuals may have different points at which they start to perceive correlations among variables or differences between two distribution means as being meaningful.

This paper will first discuss two important settings where practical significance plays a significant role: effect size interpretation and equivalence testing. Following these applications of practical significance, previous research recommendations regarding practical significance will be reviewed. Lastly, we conduct a study to explore individual perceptions of meaningfulness in visual representations of correlation and standardized mean difference.

### Interpretation of effect sizes

An effect size can be defined as the strength or magnitude of any outcome which is important to a researcher within a study’s framework or as quantitative evidence of a phenomenon’s magnitude (Cumming, 2012; Kelley & Preacher, 2012). Effect sizes and confidence intervals for the effect sizes should be reported alongside traditional null hypothesis significance testing (NHST) results to present the reader with a level of practical significance (Thompson, 2001; Cumming, 2012). Further, the smallest meaningful effect size is the recommended value to use in power analyses, in order to ensure that there is sufficient power to detect effect sizes that are potentially interesting to the researcher.

Effect sizes can be presented in unstandardized (e.g., the difference between two means) and/or standardized units. In this paper, we focus on standardized effect sizes since they can be interpreted without reference to a specific outcome variable. Cohen’s *d* (standardized mean difference) and *η*^2^ (proportion of variance explained) are commonly used effect size measures for mean differences, and *r* and *r*^2^ are commonly used measures of effect size for correlation or regression. These four measures standardize the size of the effect based on the amount of variability in the sample. However, there has been little research conducted regarding when effect sizes begin to represent a meaningful relationship. Although the effect size that represents the smallest meaningful relationship will depend on the nature of the research, understanding what individuals perceive as the smallest meaningful effect in a setting that is explicitly context-free is useful for illuminating how researchers perceive and interpret effect size. For example, researchers interpreting the magnitude of effect sizes or determining the smallest meaningful relationship for use in a power analysis will benefit from available guidelines that can be used in conjunction with the context of their study.

### Traditional difference-based testing vs. equivalence testing

Traditional NHST is used to evaluate evidence against there being no relationship among variables (e.g., differences in population means). If the researcher has a hypothesis that there is a relationship among variables, then traditional NHST is appropriate. However, when the aim of the research is to detect a lack of relationship among variables (e.g., negligible difference in means, lack of association among variables, etc.), there are alternatives to traditional difference-based NHST, like equivalence testing, that should be used to explore the absence of a relationship (e.g., Kruschke, 2011; Rogers, Howard & Vessey, 1993; Rouder et al., 2009; Wellek, 2010).

In traditional difference-based NHST, a failure to reject the null hypothesis is often wrongfully interpreted as proof of the null hypothesis (Allan & Cribbie, 2013). Since the null can never be accepted, researchers aiming to discover a lack of relationship among variables should use tests created to detect equivalence. For instance, the null hypothesis for a two-tailed equivalence test states that the magnitude of a relationship is greater than or equal to a minimum meaningful value (e.g., H_0_: ρ ≥ *δ* — ρ ≤ − *δ*), while the alternate hypothesis suggests that the magnitude of the relationship is smaller in magnitude than a minimum meaningful value (e.g., H_1_: −*δ* <ρ <*δ*).

Defining an equivalence interval (i.e., a minimum meaningful difference) before running a study is an essential step in equivalence testing, directly affecting the interpretations of findings. Rusticus & Eva (2016), Rogers and colleagues (1993), and many others, state that defining the equivalence interval relevant to a study’s context and goals can be extremely challenging. The same issues apply when researchers are adopting Bayesian analyses for the goal of providing evidence in favour of the traditional null hypothesis. For example, Kruschke (2013) outlines the “region of practical equivalence” as the Bayesian version of an equivalence interval, and Morey & Rouder (2011) discuss interval-based hypotheses for conducting Bayes Factor analyses. Accordingly, an equivalence interval which is excessively large can cause the researcher to incorrectly conclude equivalence (when there are meaningful differences among the groups), while an unreasonably small interval can cause the researcher to miss the presence of a negligible association.

Although context is extremely important when setting equivalence bounds, many general recommendations for creating these bounds exist in the literature. For example, for situations that have to do with a lack of difference among group means, guidelines have varied widely, ranging anywhere from a 5% difference (Barker et al., 2002; Rusticus & Lovato, 2011) to a 20% difference (Rogers, Howard & Vessey, 1993). Cohen’s guidelines for what constitutes a “small” relationship are also cited when researchers are exploring appropriate bounds for equivalence testing (Davidson & Cribbie, 2016). Additionally, Wellek (2010) has provided several recommendations for establishing equivalence bounds for conducting equivalence tests within a wide range of research settings. This study hopes to expand on these context-free guidelines by empirically assessing how large a relationship must be before it is determined to be meaningful, as well as how variable individuals are in their perceptions of what constitutes a meaningful relationship. Together with the context of the study, this information can assist researchers challenged with the task of setting an appropriate equivalence interval/bound.

### Suggestions regarding the smallest meaningful relationship

There are many existing recommendations for choosing the smallest meaningful relationship, such as the minimally clinically important difference (MCID) in health research. In clinical research, Fayers et al. (2000) have argued that the smallest meaningful differences used in sample size calculations should be both clinically important (e.g., to the stakeholder groups that the difference concerns) and clinically realistic (e.g., not anticipating a larger survival rate for a drug than the present rate). Methods for selecting an important or a realistic difference include (a) reviewing the current state of research about the question through meta-analysis or systematic review or (b) gathering opinions from relevant stakeholders through panels or focus groups (Cook et al., 2018).

Although these methods are ideal, they may not always be practical or feasible, especially with exploratory research. In these instances, Cook et al. (2018) state that a pilot study can be a useful way to build knowledge for what may be a realistic MCID between groups. Pilot studies are typically run in multiple phases to estimate the relevant information necessary for future, larger-scale research (Cook et al., 2014).

Additionally, there are three methods for determining an important difference which may serve as a basis for an MCID in clinical research (Cook et al., 2014). This includes the anchor method, the distribution method, and the health economic method. The anchor method uses the judgment of a relevant stakeholder to inform what an important difference may be. For instance, Jaeschke, Singer & Guyatt (1989) notes that clinicians are often able to grasp a sense of a clinically meaningful difference over time, making it reasonable to use their experience in determining what the MCID is for a clinical instrument. The distribution method suggests that the MCID may be larger than the maximum imprecision of a measurement tool (Cook et al., 2018). Distribution method approaches commonly rely upon (a) the standard error of the measurement, (b) the smallest difference detectable by a statistical test or even (c) a “rule of thumb” which focuses on a particular statistical measure (Cook et al., 2014). Health economic methods use an expected net gain to optimize the cost of a unit of a health effect with the amount that a decision maker wishes to pay, which allows researchers to quantify the expected net gain from a specific study or research protocol.

An additional way to decide upon an appropriate minimally meaningful effect, in the absence of any valuable information, is to use a standardized context-free effect size by relying on guidelines from previous research (Weber & Popova, 2012). For instance, Rogers, Howard & Vessey (1993) provide an example of a 20% difference as the smallest meaningful discrepancy between two means, while Cohen (1988) proposed a lower-bound for a standardized small effect size of *d* = 0.2 or *r* = .1. However, like Cohen, Rogers, Howard & Vessey (1993) stated that the smallest meaningful association will necessarily vary based on the research issue in question, and discouraged researchers from blindly adopting the guidelines they proposed.

Some researchers have suggested, in the context of exploratory research, that a range of possible minimum meaningful differences could be incorporated (Cribbie & Arpin-Cribbie, 2009; Weber & Popova, 2012). For instance, in clinical settings, Cribbie & Arpin-Cribbie (2009) have argued that the mean equivalence of groups may be captured by varying levels of “closeness”. Similarly, within the context of effect size research, Weber & Popova (2012) have stated that an obtained effect may be compared to a range of effect sizes to illustrate how different assumptions about an effect may influence a test decision. However, they too cautioned that their findings should be used as guidelines only when setting a minimum meaningful difference within research.

Ferguson (2009) compiled a list of suggestions for interpreting findings in the social sciences based on recommendations from previous reviews. For Cohen’s *d* and Pearson’s *r*, the minimum effect sizes representing a practically significant effect were .41 and .20, respectively.

One recent study explored the point at which individuals start to perceive the presence of a relationship. Rusticus & Eva (2016) investigated individuals’ perceptions of equivalence in order to determine an equivalence threshold for medical education data. The researchers asked participants to compare medical school training sites for their similarities on four different outcomes: undergraduate GPA, a student experience survey, a structured clinical exam, and a medical council licensing exam. The participants saw these comparisons on a series of bar graphs displaying a wide range of standardized mean differences (Cohen’s *d* from 0.10 to 1.20). The study set an equivalence threshold at the point where 50% of the participants deemed the sites to be non-equivalent. Based on their results, Rusticus and Eva established *d* = .50 as the threshold where individuals distinguish a change between distributions.

Rusticus & Eva (2016) was an example of a study where effect size interpretations were made within a prespecified context. As discussed previously, minimally meaningful effects should be utilized with a specific context in mind. Although the ideal decision about a specific meaningful effect should be made through a multi-faceted decision-making process, standardized context-free effect sizes provide helpful additional information when there are no other viable alternatives.

### The present study: quantifying a minimally important relationship

The present study aims to provide information regarding the central tendency and variability of perceptions of the importance of relationships among variables via context-free visual representations of relationships. An understanding of the magnitude and variability in interpretations of what qualifies as a meaningful relationship in context-free settings is useful for contributing to the knowledge base for researchers making decisions regarding appropriate intervals for equivalence testing, interpretations of the magnitude of effect sizes, setting the smallest meaningful effect size in power analyses, etc. in settings where context is important. The study, using a similar methodology to Rusticus & Eva (2016), asks participants to indicate whether presented figures (which either depict two overlapping group histograms or the correlation between two continuous variables) illustrate a meaningful relationship. This study also seeks to compare how perceptions of what constitutes a meaningful relationship differ across statistically-trained and statistically-untrained participants. This study will hopefully offer some first steps towards providing researchers with valuable recommendations for how to quantify when a relationship becomes meaningful; together with the specific context of the study, this information can aid researchers in understanding what constitutes a minimum meaningful association.

## Method

### Participants

This study received ethics approval from a Human Participants Review Sub-Committee (Certificate: 2017 - 001) and conformed to the Tri-Council Research Ethics guidelines of Canada. Participants were recruited using two different methods. First, 252 undergraduates taking an Introduction to Psychology course from a large Canadian university were recruited. These participants received course credit for participation in the study. We also recruited 65 graduate students and faculty from within Psychology departments across Canada via email invitation. Of these participants, 18 were enrolled in a Psychology Master’s Program, 4 indicated that they had completed a Psychology Master’s Program, 23 were enrolled in a Psychology Doctoral Program, and 16 indicated that they completed their Doctoral degree in Psychology. The sample was composed of 85 male participants and 229 female participants (three participants chose not to report their sex). Ages of the sample ranged from 18–69 with a mean and median of 22.5 years and 19 years, respectively.

To explore potential differences between statistically-trained and statistically untrained participants, all participants were asked to indicate the number of single-semester statistics courses they had taken during post-secondary education. Participants were considered to be statistically untrained if they reported completing fewer than three single-semester statistics courses and statistically trained if they reported three or more single-semester statistics courses. Accordingly, out of a total of 317 participants, 75 of the participants were considered statistically-trained, while 242 participants were considered to be statistically untrained. Generally, it is never a good idea to categorize a continuous variable, however, based upon statistical training obtained at Canadian universities, a dichotomization was created (Davidson et al., in press). In this case almost all of the undergraduate students had between zero and two statistics courses (usually, a full-year introduction to statistics course) whereas most of the remaining participants (graduate students/faculty) had significantly more than two statistics courses and therefore a dichotomization was naturally present. Specifically, for undergraduate participants: 176 of the participants stated they had taken no statistics courses, 44 stated they took one, 14 participants took two, eight took four, six took five, and only four participants took six courses or more. For graduate students and faculty: 2 participants stated they had taken one statistics course, five stated they had taken two, 19 stated they had taken three, 17 participants stated they had taken four, 11 stated they had taken five, and nine had taken six or more. Further demographic information about the sample is presented in Table 1.

**Table 1:** Demographic characteristics of the sample.

	*N*	Age (Median [IQR])	Gender, *n*	Number of Statistics Courses Taken (Mean ± SD)	Average Psychology Experience (Mode)
Statistically-trained	76	27 [10.8]	*F:* 54 *M:* 20	5.7 ± 1.8	Doctorate Ongoing
Statistically-untrained	241	19 [2.0]	*F:* 175 *M:* 65	1.3 ± 0.6	First Year Undergraduate Ongoing
Overall	317	19 [5.0]	*F:* 229 *M:* 85	2.4 ± 2.1	First Year Undergraduate Ongoing

**Notes.**

F female M male

Exclusion criteria were created to screen for inattentive responding, difficulty comprehending the task, etc. Participants were excluded from the study if they responded incorrectly to four or more out of a possible thirteen screening questions. The screening questions included four example questions that provided participants with instructions for the survey; these sample questions explicitly directed the participants towards selecting the correct option. The other nine questions used as part of the exclusion criteria included extreme examples of associations or lack of associations among variables from the study. More specifically, the questions from the study that were used in screening for inattentiveness included: (a) a very large standardized mean difference (overlapping histograms depicting a Cohen’s *d* of 1.95 or 2.00), (b) zero/minimal standardized mean difference (overlapping histograms depicting a Cohen’s *d* of 0.00 or .05), (c) a very large correlation among variables (scatterplots depicting correlations of .60 or −.60), and (d) zero/minimal correlation among variables (scatterplots depicting correlations of −.05, 0.00, and .05). Based on these screening questions, 105 participants were omitted from data analysis, leaving a total of 212 participants (54 in the statistically trained group and 158 in the statistically-untrained group).

### Measures

First, participants were presented with figures displaying varying degrees of relationships and were asked to classify the relationships as meaningful or not. The word “meaningful” was used in our measures to encourage participants to assess the magnitude of difference or relationship within a figure. Two different types of figures were shown: (1) Side-by-side histograms; and (2) Scatterplots.

**Figure 1:**
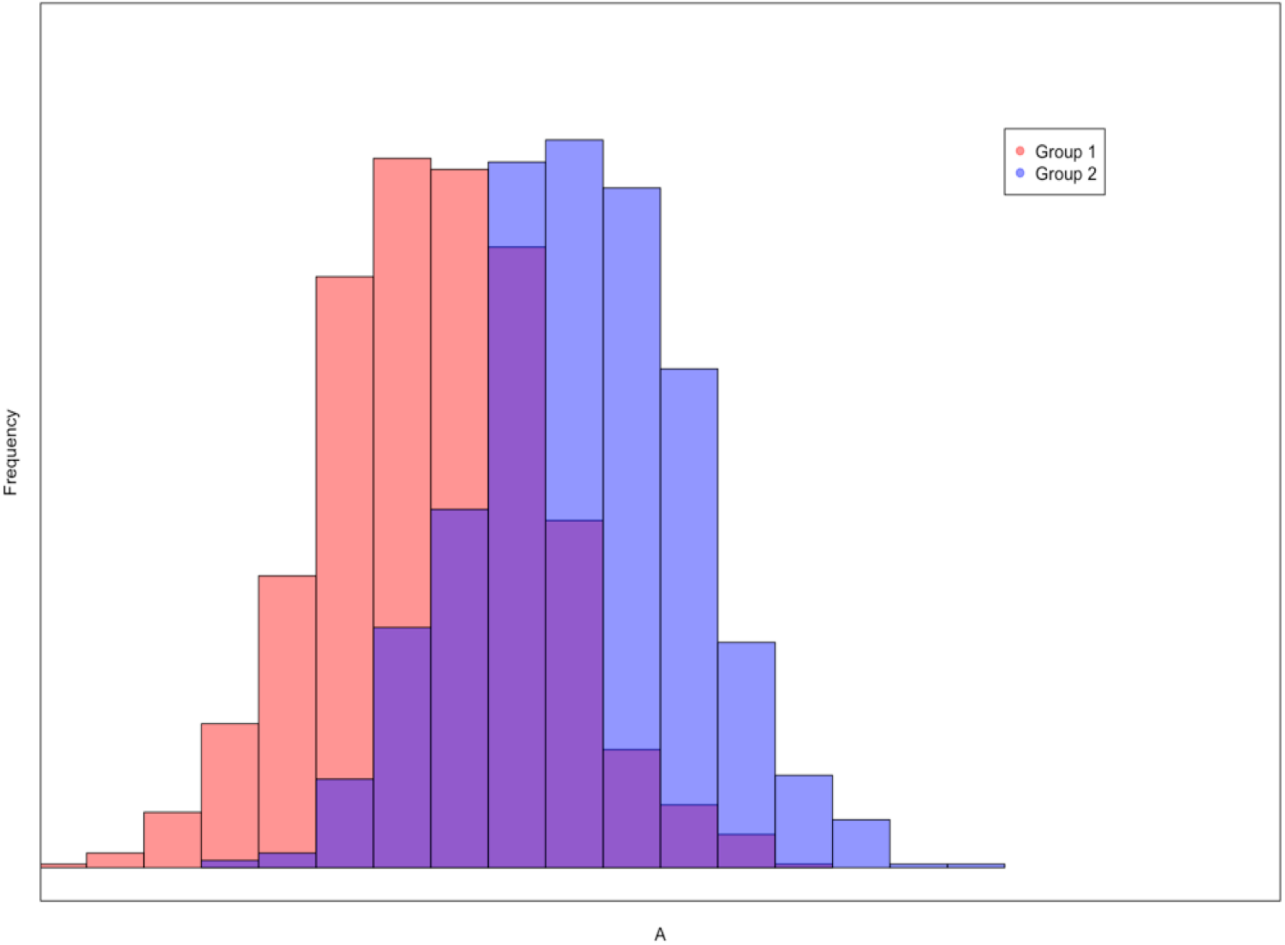
An example of an overlapping histogram seen by the participants. The red distribution represents the frequency of scores from Group 1, the blue distribution represents the frequency of scores from Group 2, and the purple coloring indicates where the distributions overlap. In this example Cohen’s *d* = 1.3.

The histograms displayed two distributions that were separated by a population Cohen’s *d* ranging from 0.00 to 2.00, in .05 increments (41 total side-by-side histograms), with a sample size per group of *N* = 1,000. The first histogram in each pair had a population mean of 0, while the second had a population mean set at each Cohen’s *d* value explored. Distribution shapes were normal and all had a population standard deviation of one. These figures were generated using the *hist* function from the *graphics* package in R (R Core Team, 2016). For each figure, participants were asked to choose between one of two alternatives: (1) *There is a meaningful difference between the scores of the groups*; or (2) *There is not a meaningful difference between the scores of the groups*. An example of a histogram figure seen by the participants is presented in Fig. 1.

**Figure 2:**
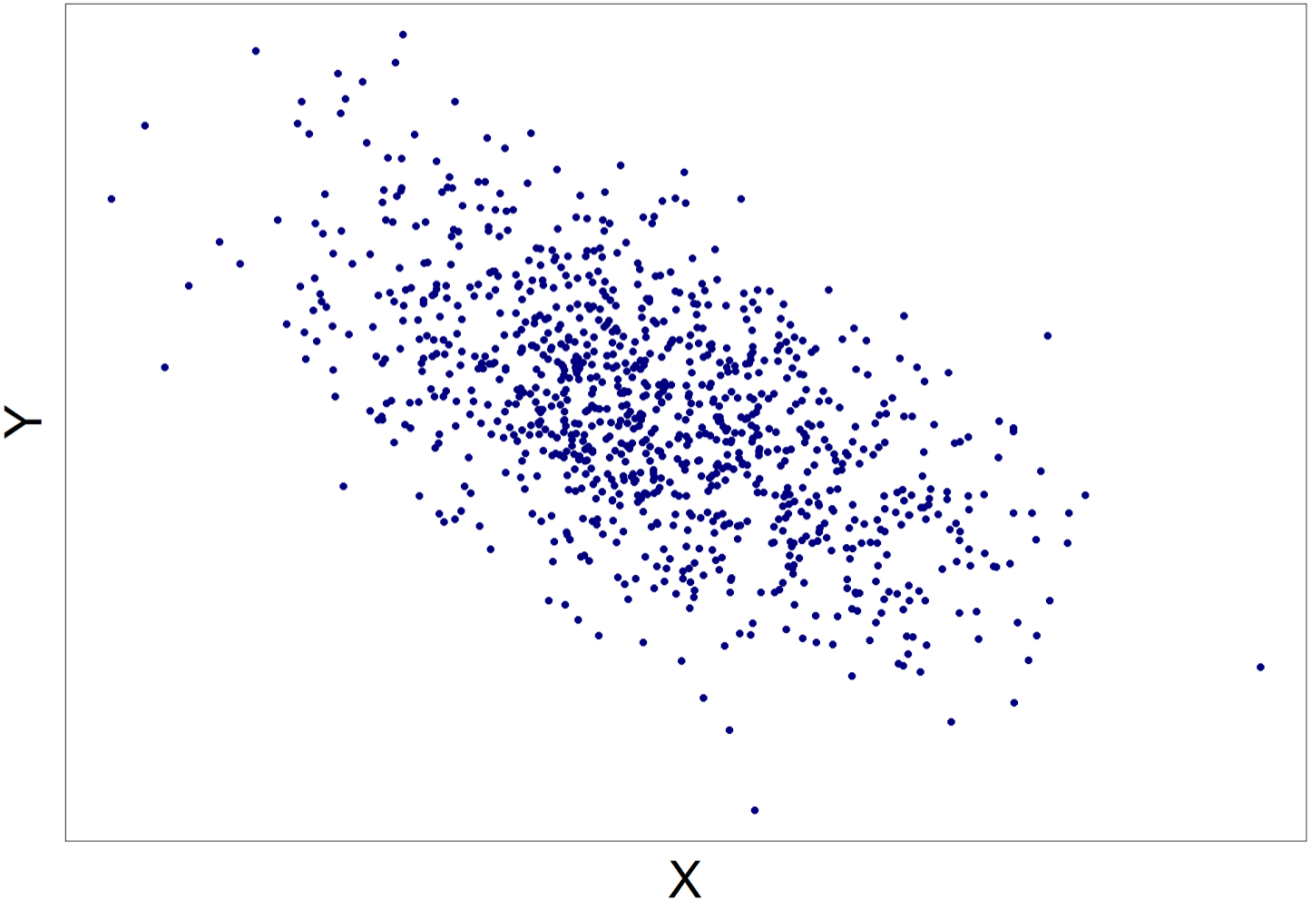
An example of a scatterplot seen by the participants. In this case *ρ* =  − .55.

The scatterplots displayed population correlations ranging from −.60 to .60 in .05 increments (25 total scatterplots), with a sample size of *N* = 1,000. These figures were generated using the *plot* function within the *graphics* package in R (R Core Team, 2016). In each instance, *X* and *Y* variables were sampled from a bivariate normal distribution with means of zero and standard deviations of one. For each scatterplot, participants were asked to choose between one of two alternatives: (1) *There is a meaningful relationship between the variables X and Y*; or (2) *There is not a meaningful relationship between the variables X* and *Y*. An example scatterplot is presented in Fig. 2.

In order to ensure that individual figures were not contaminated by outliers, specific patterns of cases, etc., five figures were generated for each effect size (i.e., each value of *d* and *r*) and participants were randomly assigned one of the five figures for each of the effect size conditions.

### Procedure

All participants accessed the survey using SurveyMonkey online software (http://www.surveymonkey.com). After providing informed consent regarding their participation, participants were asked for demographic information.

**Figure 3:**
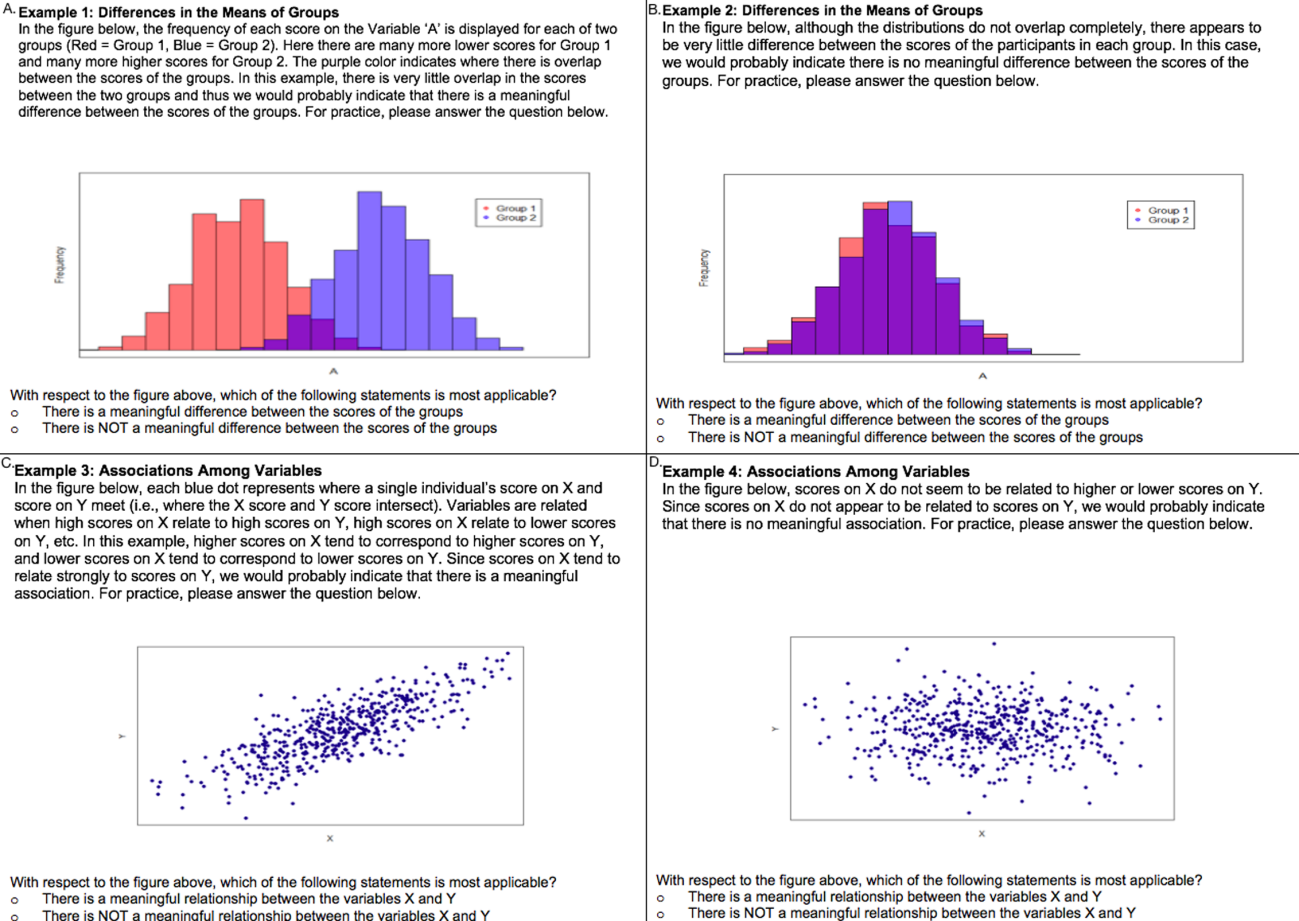
Example questions shown to participants before beginning the study. Specifically, (A) depicts a difference in group means with distributions that have very little overlap, (B) depicts a difference in group means with distributions that have considerable overlap, (C) depicts an association between variables where X and Y appear to be strongly associated, and (D) depicts an association between variables where X and Y do not appear to be strongly associated.

Participants were then shown four examples of the types of figures that they would encounter throughout the remainder of the survey. The two histogram examples depicted distributions of scores for two groups that had (1) very little overlap and (2) almost complete overlap. Similarly, the example scatterplots showed participants (1) a nearly perfect correlation and (2) zero correlation. Each of the examples also contained a paragraph describing the figure, drawing attention to relevant features of the graphs (i.e., overlap in the case of histograms or the association between *X* and *Y* scores in the case of scatterplots). In other words, the participants were provided with brief introductory training on how to interpret the side-by-side histograms and scatterplots. In order to ensure that participants were able to accurately interpret the displayed relationships, participants were asked to decide whether or not each example illustrated a meaningful difference (histograms) or meaningful association (scatterplots). Figure 3 displays the four sample figures along with the training material and questions answered by participants.

After viewing the examples, participants were shown the overlapping histograms and scatterplots and, similar to the introductory training, were asked to rate each one as displaying either a meaningful or non-meaningful standardized mean difference or correlation, respectively. These questions were identical to the four examples in the introductory training, however, no instructions above the figures were provided. The order of presentation of the figures was random to control for any potential order bias. Participants could move back and forth through the survey as desired and even return to the introductory training figures that were preceded with explanations.

## Results

The results below are based upon the data provided by the 212 participants who passed the exclusion criteria. The findings from our analysis were largely similar regardless of whether the entire sample’s data or the 212 participants’ data was used.

### Cohen’s *d*

An algorithm was created to quantify each individual’s unique threshold for detecting when the scores of the two groups became meaningfully different from one another. Each participant’s cut-off point was defined as the midpoint between (1) the maximum two consecutive effect sizes where the participant indicated that the scores of the groups were not meaningfully different and (2) the minimum two consecutive effect sizes where the participant indicated that the scores of the groups were meaningfully different. For example, suppose a participant indicated that the scores of the groups were not different for the last time consecutively at a Cohen’s *d* of 0.75 and 0.80, then judged the groups’ scores as different for the first time consecutively at a Cohen’s *d* of 0.85 and 0.90. Accordingly, the participant’s cut off point would have been estimated at a Cohen’s *d* of 0.825 (mean of 0.80 and 0.85).

The unique cut-off points for the statistically-untrained and statistically-trained groups were both approximately normally distributed. The range of unique cut-offs varied from *d* = 0.22 to *d* = 1.73 for the 156 statistically-untrained participants (note that cut-off points could not be calculated for two statistically-trained and two statistically-untrained participants since the algorithm required a particular pattern of answers that was not present in these participants’ responses). Statistically untrained participants’ cut-off points had a mean of *d* = 0.95 (SD = 0.30), 95% CI [0.36, 1.54]. For the 52 statistically-trained participants, the range of unique cut-offs varied from *d* = 0.38 to *d* = 1.92. Trained participants had a mean cut-off of *d* = 1.21 (*SD* = 0.42), 95% CI [0.41, 2.01]. It is noteworthy that there is significant variability across participants in terms of what constitutes a meaningful relationship and that no participant had a cut-off below what Cohen (1988) labeled a “small” effect (i.e., *d* = .2).

A hierarchical logistic regression was run using the *glmer* function from the *lme4* package in R (Bates et al., 2015). Participants’ binary response to whether two histograms were meaningfully different was the outcome variable of the model. The model’s predictors were the Cohen’s *d* values (between 0.00 and 2.00), age, participants’ level of statistical training, and the interaction between the Cohen’s *d* value and statistical training.

**Figure 4:**
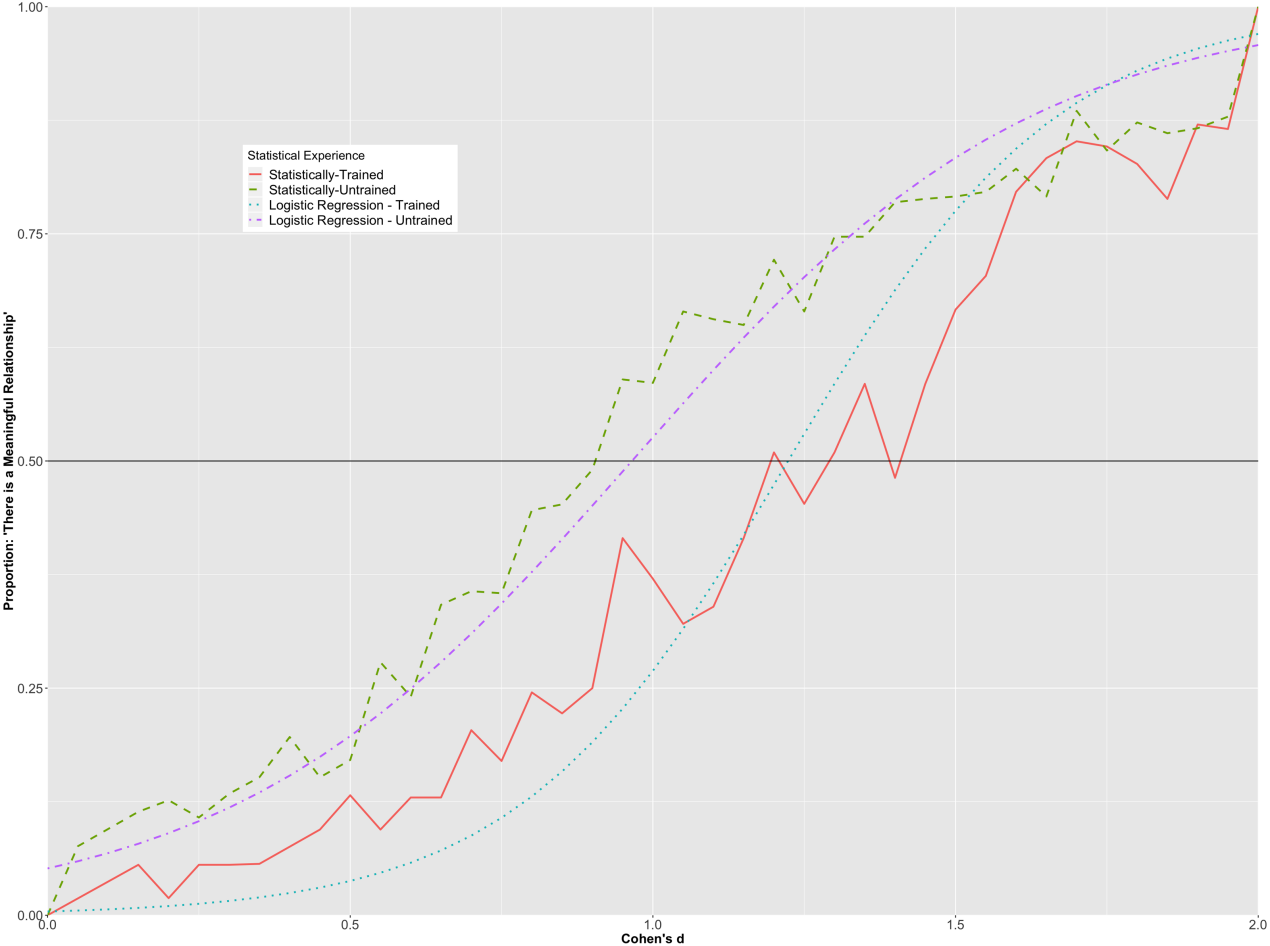
Hierarchical/mixed logistic regression for Cohen’s d. Graph depicts the predicted probabilities of statistically-trained and statistically-untrained participants stating that there is a meaningful mean difference between groups, plotted alongside the observed proportion for each Cohen’s *d* value.

The overall prediction accuracy of the model was 85% with a pseudo *R*^2^ of .46 (Nakagawa & Schielzeth, 2013). A statistically significant interaction was observed between Cohen’s *d* values and participants’ statistical training (*p* < .001). To better understand the nature of the interaction, the predicted probabilities for each group at each level of Cohen’s *d* were plotted in Fig. 4, along with the observed proportions for each of the Cohen’s *d* values in each of the groups. Figure 4 suggests that participants with greater statistical training required a larger Cohen’s *d* than participants with less statistical training before indicating that the difference in the scores of the groups was meaningful. For example, although the probabilities were similar at small and large values of Cohen’s *d*, the statistically-trained group had a greater than 50% probability of indicating that there was a meaningful relationship when Cohen’s *d* reached 1.00, whereas the statistically-trained group had a greater than 50% probability of stating that there was a meaningful relationship when Cohen’s *d* reached 1.25. These values were almost identical to those computed using the raw proportions for each value of Cohen’s *d*.

To further investigate the interaction between participants’ statistical training and the different Cohen’s *d* values on whether or not a standardized mean difference was considered meaningful, we consider both groups’ relative likelihood of indicating that there was a meaningful difference between the groups at different Cohen’s *d* values. For example, at a Cohen’s *d* of .25, a participant in the statistically-trained group had a 1.3% predicted probability of stating that there was a meaningful difference between groups, while a participant in the statistically-untrained group had a 10.4% predicted probability. Further, given a Cohen’s *d* of .90, a statistically-trained and a statistically-untrained participant had a 19.2% and a 45.1% likelihood of claiming that there was a meaningful difference between the groups, respectively. Finally, at a Cohen’s *d* of 1.5, statistically-trained and statistically-untrained participants had a 77.5% and an 83.4% chance of stating there was a meaningful difference between histograms, respectively. As expected, there was very little difference between the two groups at the extremes of Cohen’s *d* (*d* < .10, *d* >1.60).

### Correlation

Similar to Cohen’s *d*, an algorithm was utilized to investigate each participant’s unique judgment point for indicating when the variables *X* and *Y* are related to one another. Since the graphs depicted scatterplots with both negative and positive correlations, the absolute value of the correlations was utilized for the calculation of each participant’s unique cut-off. The same criteria that was used with the standardized mean differences was also applied in the correlation setting.

The statistically-untrained and statistically-trained groups’ cut off points were both approximately normally distributed. When participants had opposing responses to correlation values in positive and negative directions (e.g., noting variables were meaningfully associated for ρ = 0.30 but not meaningfully associated for ρ =  − .30) we randomly assigned a meaningfully associated or not meaningfully associated response. For the 140 statistically-untrained participants (again, cut-offs were not able to be estimated for seventeen statistically-untrained and nine statistically-trained participants because their responses did not match the pattern required by the algorithm), cut-offs ranged from ρ = 0.08 to ρ = 0.52, with a mean of ρ = 0.32 (*SD* = 0.10), 95% CI [0.13, 0.49]. For 53 statistically-trained participants, cut-offs ranged from 0.12 to 0.52, with a mean of ρ = 0.28 (*SD* = 0.08), 95% CI [0.10, 0.46].

As with Cohen’s *d*, a hierarchical logistic regression was run using the *glmer* function. Participants’ binary response to whether two variables were meaningfully associated was the outcome variable of the model. The model’s predictors were the correlation values (ρ values from 0 to .60, by .05, recalling that we took the absolute value of the negative correlations), participants’ level of statistical training and age, and the interaction between the ρ values and level of statistical training.

**Figure 5:**
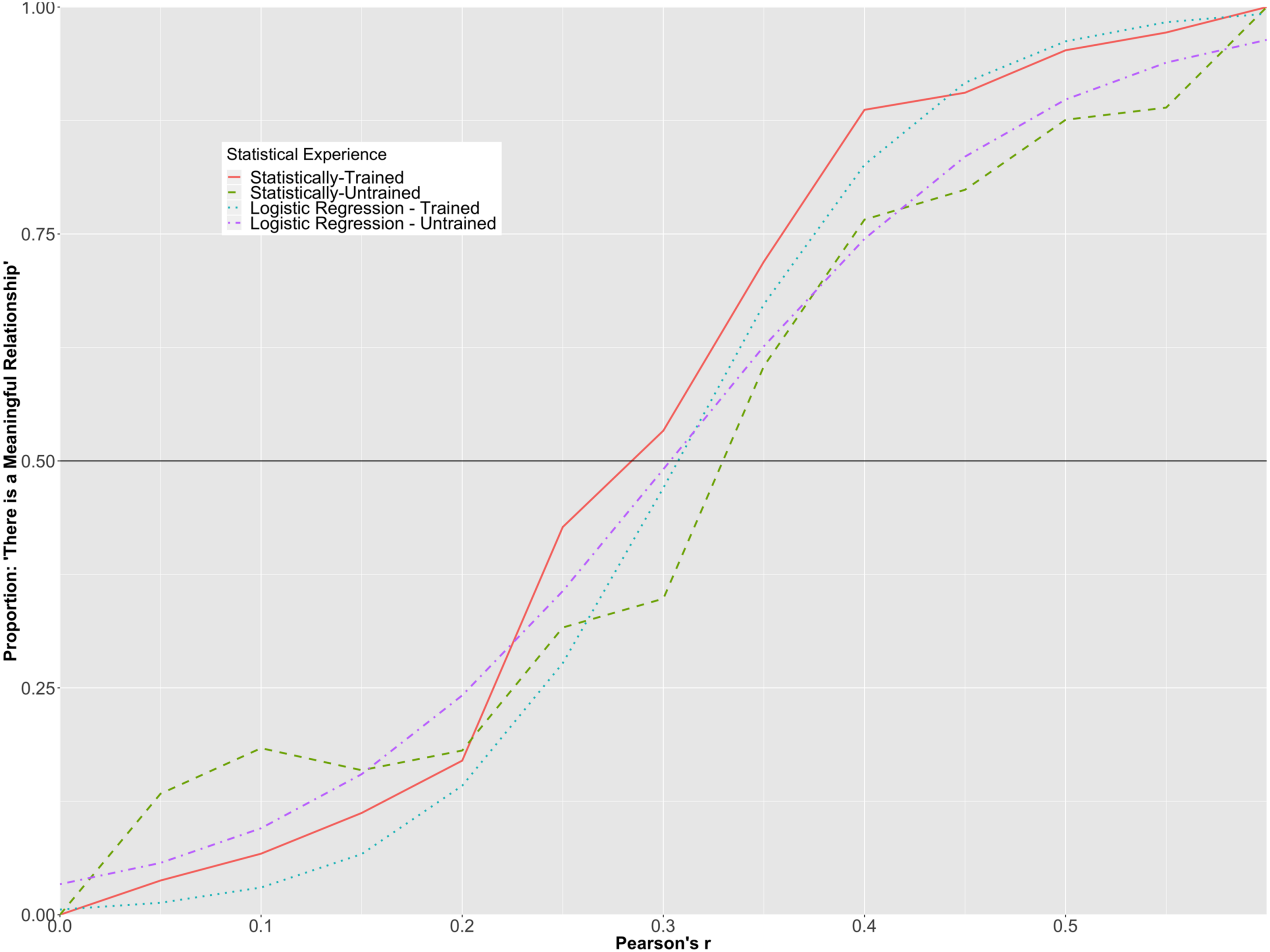
Hierarchical/mixed logistic regression for Pearson’s r. This graph depicts the predicted probability of statistically-trained and statistically-untrained participants stating that there is a meaningful relationship among two variables, plotted alongside the observed proportion for each Pearson’s *r* value.

The overall prediction accuracy of the model was 77%, with a pseudo *R*^2^ = .55 (Nakagawa & Schielzeth, 2013). Like the model representing Cohen’s *d*, there was a statistically significant interaction between correlation strength and participants’ level of statistical training (*p* < .001). To further illuminate upon the interaction, the predicted probabilities for each group at each level of correlation strength were plotted in Fig. 5 along with the observed proportions for each of the correlation values in each of the groups.

Figure 5 suggests that participants with more statistical training had lower predicted probabilities of perceiving smaller correlations as meaningful than those with less statistical training, whereas they had higher predicted probabilities of perceiving larger correlations as meaningful than those with less statistical training. However, it is clear from the graph that the magnitude of the interaction was not as strong as that found for standardized mean differences and the vast majority of the variability explained can be attributed to the differences in the population correlations. For example, the statistically-trained group had a greater than 50% probability of indicating that there was a meaningful association between variables when Pearson’s ρ was .30, while the statistically-trained participants had a greater than 50% probability of stating that there was a meaningful relationship when Pearson’s ρ was .35.

When comparing both groups’ relative likelihood of indicating that there was a meaningful association among the variables, it was found that while a person in the statistically-untrained group was more likely to indicate that there was a meaningful association between variables at ρ = .15 (15.5% probability compared to 6.7% for the statistically-trained participant), both groups had a relatively equal probability of stating that there was a meaningful association between variables at ρ = .25 (35.1% for the inexperienced participant and 27.7% for the experienced participant). At ρ = .40, a statistically-trained participant had a notably greater likelihood than a statistically-untrained participant of claiming that there was an association between variables (82.6% vs 60.1%, respectively).

When we investigated the data for negative correlation values separately from the data in the positive direction, we found similar findings for both the logistic model and the proportions of participants that indicated the variables were meaningfully associated.

## Discussion

This study explored how large a graphical depiction of an association among variables must be in order for individuals to interpret the relationship as meaningful. The goal of the study was to be able to assist researchers conducting equivalence testing, interpreting the magnitude of effect sizes, setting the smallest meaningful effect in power analyses, etc. in exploring when individuals perceive a minimally important relationship in a context-free setting. In the past, when researchers are looking for generic (i.e., context-free) guidelines for interpreting the magnitude of effect sizes, many have relied upon Cohen’s suggestions for small, medium and large effects, however there has been an almost complete lack of empirical research exploring when individuals begin to detect a meaningful relationship. This investigation utilized graphical depictions of standardized mean differences and correlations and asked participants to indicate for several different degrees of relationship whether or not the association was meaningful. More specifically, participants viewed overlapping histograms and scatterplots displaying varying degrees of effect size and indicated which relationships were meaningful.

When participants viewed overlapping histograms displaying effect sizes ranging from *d* = 0.00 to *d* = 2.00, individual cut-off points varied from *d* = 0.22 to *d* = 1.73 and had a mean of *d* = 0.95 for statistically-untrained participants. For statistically-trained participants, Cohen’s *d* individual cut-offs were between *d* = 0.38 and *d* = 1.92 and had mean of *d* = 1.21. When participants were shown scatterplots depicting the relationship between two variables (ranging from ρ = 0 to ρ = .60, including both positive and negative correlations), individual cut-off points for both statistically-untrained participants and statistically-trained participants ranged from ρ = 0.12 to ρ = 0.52, with statistically-trained participants having a mean cut-off point of ρ = 0.28 and statistically-untrained participants having a mean of ρ = 0.32.

Accordingly, in a context free setting, our study indicates that larger effect sizes than expected are required for participants to state that relationships among variables or differences between groups are meaningful. We say “than expected” since it has been common in the behavioural sciences to label *r* = .1/ *d* = .2 a small effect and *r* = .3/ *d* = .5 a moderate-sized effect (Cohen, 1988; Nye et al., 2018). As noted by Rosenthal & Rosnow (1984), a Pearson’s *r* equivalent to the Cohen’s *d* large effect (*d* = 0.80) is *r* = 0.31 (p. 361). Interestingly, on average, participants in our study began to judge associations as meaningful around these large cut-offs. Intriguingly, Szucs & Ioannidis (2017) noted that the 25th and the 75th percentile for effect sizes in psychology is *d* = (0.29, 0.96). Thus, comparing the results of our study to these past recommendations/results, there appears to be quite a disconnect between what participants in our study are deeming to be the minimally meaningful effect size and both recommendations for declaring an effect meaningful and observed effect sizes. Given the preliminary nature of this research, and the fact that we explored context-free relationships, we are not contending that most findings in the behavioral sciences are meaningless. However, these results definitely suggest that more research is required into how large an effect must be before it can be considered meaningful; our results reinforce, as has always been the case, that blind adoption of popular cut-offs for effect sizes is not recommended. Thus, the results of this study, along with the many perspectives that may be used to obtain an understanding of when an effect is meaningful, can be valuable to researchers faced with the extremely difficult task of quantifying when an association becomes important.

The interaction between the *d* values and level of statistical training was an interesting finding. Participants with more statistical training needed a larger standardized mean difference before interpreting the relationship as meaningful. One potential cause of the difference is that those taking more statistics courses (i.e., graduate students and faculty members) have also had more experience assessing what level of relationship is meaningful (or not) within their own research programs, but it is unclear why the statistically-trained group would require a larger effect before declaring the relationship meaningful. The interaction between the Pearson’s ρ values and the level of statistical training highlighted that although there was very little difference among the groups at small or very large correlations in terms of what constituted a meaningful relationship, more participants in the statistically trained group tended to declare correlations between ρ = .35 and ρ = .50 meaningful than their statistically untrained counterparts. The effect, as evidenced in Fig. 5, was not large. One explanation for our findings regarding the effect of level of statistical training could relate to interpretational difficulties on the part of the statistically-trained group (e.g., Allen, Dorozenko & Roberts, 2016; Lem et al., 2014a). For example, in some instances, statistically-trained participants may be more likely to focus upon the structural/analytic, as opposed to surface, components of figures (Lem et al., 2014b). In our study, focus upon such structural features would include a consideration of the overlap created by two side-by-side histograms. In contrast, those who have less statistical training may tend to focus on more surface-level features of the figures, such as the height of the bars of the histograms. It is worth noting that even though our survey did not have a time limit, if any participant felt overwhelmed or rushed during the survey, they could have been more prone to looking at surface level features. Accordingly, more research is required into the cause(s) of the observed interaction effects.

## Conclusions

The results presented here, along with previous findings regarding the interpretations of the magnitudes of relationships, and, most importantly, the context of the study, can be used to make judgements regarding when a relationship becomes meaningful. As noted by Cohen (1988), the interpretation of effect sizes in final analyses is dependent upon the environment in which it is embedded. Accordingly, no guidelines can replace a researcher’s expertise about their study at hand. To put this in perspective, currently many researchers utilize information such as Cohen’s (1988) cut-offs in conjunction with the context of the study to make judgments regarding the magnitude of an effect. It is hoped that the results gleaned from this study can also be used by researchers to better understand the magnitude of an effect. These strategies are already being implemented in areas such as oncological research, where graphs of standardized effect sizes, such as forest plots, are used to enhance interpretations of study findings (Bell et al., 2017).

There are a few limitations of this study that should be highlighted. First, differences between the statistically trained and untrained participants need to be treated with caution since there were only 54 participants in the statistically trained group and therefore estimates of those parameters are not as precise as desired. Second, for participants with little or no experience in statistics, the training at the beginning of the survey may not have been sufficient to ensure they were comfortable completing the survey. Further, it was necessary to assign participants a random response when they had opposing responses between negative and positive directions about the relation between *X* and *Y* variables for any given ρ. Doing this allowed for more participants’ cut-off points to be estimated, but also meant that at times it was not possible to incorporate each participant’s precise pattern of responding into the calculation. It is also worth noting that the inexperienced sample came from a university participant pool and may not be representative of the broader pool of inexperienced participants such as the general population. Similarly, although we used the number of statistical courses taken by participants to measure statistical training, other factors that were not recorded could have also affected statistical training and experience such as the grades received in the courses, the course level and course credit value. Finally, we only investigated the question of what constitutes a meaningful relationship in two-group standardized mean difference and bivariate correlation settings. Further research will hopefully extend these results into other popular research designs.

To summarize, our study provides information that can be used alongside contextual factors to help researchers interpret effect sizes or determine what constitutes the smallest meaningful association (e.g., for setting bounds in equivalence testing or conducting a power analysis).

## Additional Information and Declarations

### Data Availability

The following information was supplied regarding data availability: Data is available at the Open Science Framework: https://osf.io/wut42/?view_only=ae5fd48ed18c4868b2b269cdb0045d28.
